# Prognostic significance of inflammatory and nutritional markers in perioperative period for patients with advanced gastric cancer

**DOI:** 10.1186/s12885-022-10479-6

**Published:** 2023-01-03

**Authors:** Ruolan Zhang, Can Hu, Jiaqing Zhang, Yanqiang Zhang, Li Yuan, Pengcheng Yu, Yi Wang, Zhehan Bao, Mengxuan Cao, Rongwei Ruan, Xiangdong Cheng, Zhiyuan Xu

**Affiliations:** 1 The Second School of Clinical Medicine of Zhejiang Chinese Medical University, Hangzhou, 310053 China; 2 The Cancer Hospital of the University of Chinese Academy of Sciences (Zhejiang Cancer Hospital), Institutes of Basic Medicine and Cancer (IBMC), Chinese Academy of Sciences, Hangzhou, 310022 China; 3 The First School of Clinical Medicine of Zhejiang Chinese Medical University, Hangzhou, 310053 China; 4 Wenzhou Medical University, Wenzhou, 325035 China

**Keywords:** Stomach neoplasms, Neoadjuvant therapy, Inflammation, Prognosis

## Abstract

**Background:**

It has been reported that inflammatory and nutritional markers are related to prognosis in numerous malignancies. The present study analyzed the significance of these markers’ alterations during neoadjuvant chemotherapy in the long-term outcomes in patients with advanced gastric cancer.

**Methods:**

A retrospective review was performed of 437 advanced gastric cancer patients who underwent a neoadjuvant chemotherapy (NACT) regimen followed by surgical treatment. Inflammatory and nutritional markers measured from the blood samples collected from the patients before the first neoadjuvant chemotherapy and after the last neoadjuvant chemotherapy were used for analysis. Statistical analysis, including Mann-Whitney U or chi-square tests, the Kaplan-Meier method and Cox multivariate analysis, were performed to analyze the predictive value of these markers for overall survival outcomes (OS).

**Results:**

Most biomarkers, including lymphocyte, leucocyte, neutrophil, monocyte, platelet, LMR, PLR, SII, CRP, CAR, hemoglobulin and albumin levels, changed during NACT (*P* <  0.05). After separately grouping the patients based on the normal range of hematologic indexes and the change rate (α) of systemic inflammatory and nutritional markers by the cutoff value derived from X-tile (P <  0.05), we found that differentiation, TRG, pre-NACT BMI, pre-NACT platelet counts, post-NACT lymphocyte counts, the change in lymphocyte counts, change in platelet counts and LMR(α), PLR(α), SII(α), and CAR(α) were associated with OS. Multivariate analysis revealed that PLR (α) > − 19% was correlated with a 3.193-fold (95% CI: 2.194–4.649) higher risk of death (*P* <  0.001) than others.

**Conclusion:**

NACT could significantly change several inflammatory and nutritional markers in the perioperative period; the platelet counts before NACT, and the change in lymphocytes during NACT truly correlated with long-term outcomes among patients with advanced gastric cancer. The systemic inflammatory marker PLR may be a reliable marker for the prediction of prognosis.

**Supplementary Information:**

The online version contains supplementary material available at 10.1186/s12885-022-10479-6.

## Background

Gastric cancer (GC) remains a serious cancer worldwide and have caused more than one million new cases in 2020 and approximately 769,000 deaths, which is equal to one in every 13 deaths globally, and it ranks the fifth for morbidity and the fourth for mortality globally [1]. Most patients were diagnosed at advanced stage, especially in China [2]. A growing number of clinical studies have verified that perioperative/neoadjuvant chemotherapy (NACT) benefits survival more than surgery alone in the treatment of advanced GC [3]. NACT could not only delay local tumor progression partly but also contribute to distinguishing patients who could not gain a survival benefit from surgery due to disease development during neoadjuvant therapy [4]. Surgery and systemic chemotherapy are recognized internationally as standard treatments for patients with advanced GC [4, 5].

In Asian countries, perioperative chemotherapy prior to radical gastrectomy has also improved tumor remission rates and radical (R0) resection [2]. Therefore, for feasibly resectable patients whose clinical Tumor Node Metastasis (TNM) score is higher than T2N0, NACT is typically administered rather than postoperative adjuvant chemotherapy [4]. However, survival benefits of perioperative chemotherapy compared with postoperative chemotherapy for radical D2 gastrectomy should still be confirmed by larger phase III clinical trials such as RESOLVE study [6]. Because of tumor heterogeneity, patients with the same TNM stage or the same therapy may have different outcomes. Furthermore, no clinical guidelines exist on the number of cycles of perioperative chemotherapy and the optimum timing to surgery after NACT. Thus, it is necessary to screen out patients who are fit for perioperative chemotherapy at an early stage and monitor performance during perioperative chemotherapy.

As researchers have delved into the tumor immune microenvironment (TIME) more deeply in recent years, the finding that the occurrence and progression of tumors could be influenced by the systemic inflammatory response is increasingly recognized [7–9]. The OS outcomes of cancer patients was significantly associated with their nutritional state, especially for those who would accept chemotherapy or undergo an operation [10]. Recently, several studies have claimed that inflammatory and nutritional markers are independent prognostic indicators in some malignancies, including GC [11].

Nevertheless, most published studies have focused only on the values of these biomarkers before or after NACT, while the prognostic value of pre-neoadjuvant chemotherapy (pre-NACT) and post-neoadjuvant chemotherapy (post-NACT) marker changes remain imprecise. A method that could reliably predict prognosis to prevent wasting time in the perioperative period and improve surgical outcomes is required. The aims of this study were to conduct a complete analysis of inflammatory and nutritional markers in advanced GC patients undergoing gastrectomy after NACT and to investigate the changes in marker correlations with survival. The present study used retrospective clinical trial data to explore whether several inflammatory and nutritional markers could be effective predictors of OS.

## Materials and methods

### Patients

We retrospectively enrolled 437 patients who were diagnosed with primary gastric cancer and underwent gastrectomy after neoadjuvant chemotherapy from March 2010 to September 2020 in Zhejiang Cancer Hospital in China. The inclusion criteria were as follows: (1) histologically confirmed gastric cancer; (2) received surgical treatment after neoadjuvant chemotherapy; and (3) measurement of serum inflammatory and nutritional markers before and after neoadjuvant chemotherapy. (4) underwent a total or subtotal gastrectomy; and (5) had complete medical records; The exclusion criteria were as follows: (1) any pretreatments (radiotherapy and neoadjuvant treatments other than chemotherapy); (2) other malignancies; (3) other organ insufficiencies; and (4) acute events within the last three months (cerebral, coronary, and so forth). The basic and clinicopathological features of these patients, including sex, age, tumor location, differentiation, neoadjuvant chemotherapy cycles and responses, and survival, were reviewed.

Pathological response to chemotherapy was assessed using the tumor regression grade (TRG) system, which is defined by the American Joint Committee on Cancer (AJCC) Cancer Staging Manual, 8th edition: 0 (no residual cancer cell), 1 (single cells or small groups of cells), 2 (residual cancer with desmoplastic response), or 3 (minimal evidence of tumor response) [12, 13]. According to the TRG, we divided the patients into 2 groups: (1) tumor regression: TRG0 and TRG1; (2) tumor residue: TRG2 and TRG3. The characteristics of the operation, such as the surgical method (open or laparoscope) and the type of resection (proximate, distal, total gastrectomy), were also collected. by specialist gastrointestinal pathologists as part of our standard pathological assessment of the postoperative specimen, developed by Mandard et al. The pathologic responses to neoadjuvant chemotherapy were determined by the criteria defined by the MD Anderson center and according to the histological examination post-operation. Regarding survival time, all patients were periodically followed up in outpatient visits and telephone interviews after surgery. OS was the primary endpoint, and the survival time was calculated from the date of first neoadjuvant chemotherapy to the date of death or the last follow-up visit (follow-up for 48 months).

### Data management

Inflammatory and nutritional markers are markers which can reflect patients’ infection and nutritional status. And leucocyte level, neutrophil level, lymphocyte level, monocyte level, platelet level, C-reactive protein (CRP) level are inflammatory markers. Hemoglobulin concentration, albumin level and body mass index (BMI) could reflect the nutritional of patients.

The inflammatory and nutritional markers were calculated or rated at two time points, namely, the timing of pre-NACT and the timing of post-NACT, based on the peripheral blood tests in the database from Zhejiang Cancer Hospital. The blood test before NACT was performed at the first visit, while the blood test after NACT was performed one month after the last chemotherapy and just before the operation. No patients were administered granulocyte-colony stimulating factor during NACT, which would affect the immuno-inflammatory markers. Leucocyte level, neutrophil level, lymphocyte level, monocyte level, platelet level, hemoglobulin concentration, albumin level, C-reactive protein (CRP) level, neutrophil-to-lymphocyte ratio (NLR), lymphocyte-to-monocyte ratio (LMR), platelet-to-lymphocyte ratio (PLR), systemic immune-inflammation index (SII), C-reactive protein-to-albumin ratio (CAR), hemoglobulin concentration, serum albumin, and body mass index (BMI) were collected and calculated based on the peripheral blood tests in the database from Zhejiang Cancer Hospital. The NLR was defined as the absolute neutrophil level divided by the absolute lymphocyte level. The LMR was defined as the absolute lymphocyte level divided by the absolute monocyte level. The PLR was defined as the absolute platelet level divided by the absolute lymphocyte level. The CAR was calculated by dividing the serum CRP level by the serum albumin level. The SII was defined as the absolute platelet level multiplied by the absolute neutrophil level and then divided by the lymphocyte level. Moreover, the changes in inflammatory and nutritional markers before and after NACT were labeled the change rate (α):

$$\alpha=\text{post}-\text{NACT}\;\text{markers}/\text{pre}-\text{NACT}\;\text{markers}-1$$

The inflammatory and nutritional markers are divided into different groups based on hematologic indexes (leucocyte level, hemoglobulin concentration, neutrophil level, lymphocyte level, monocyte level, platelet level, CRP level, albumin level) and systemic inflammatory and nutritional markers (NLR, LMR, PLR, CAR, SII, BMI). Although BMI is systemic nutritional marker, it also has a clinically normal range like basic hematologic indexes. Therefore, we assigned it to ‘hematologic indexes’ group in follow-up analysis because of its particularity.

According to the standard of Zhejiang Cancer Hospital’s clinical laboratory of hematologic indexes, Leucocyte [3.5–9.5], Hemoglobulin [13–17.5], Neutrophil [1.8–6.3], Lymphocyte [1.1–3.2], Monocyte [0.1–0.6], Platelet [125–350], CRP [0–10], Albumin [40–50], and BMI [18.5–24.9], the patients were divided into 2 or 3 groups: normal/abnormal or below/normal/abnormal. Then, survival analysis was used to evaluate the pre-NACT and post-NACT data. Similarly, we grouped the patients by the cutoff value derived from X-tile of the change rate (α) of systemic inflammatory and nutritional markers [14]. We also divided the patients into groups according to the changes in hematologic indexes. Up-regulated: the index was below the normal level before NACT and then changed to normal or above normal after NACT, or the index was normal before NACT and then changed to above normal after NACT. Non-regulated: the index remained at the same level during NACT. Down-regulated: the index was above the normal level before NACT and then changed to normal or below normal after NACT, or the index was normal before NACT and then changed to below normal after NACT.

Finally, we performed survival analysis based on the aforementioned groups and then verified the combination of the predictive value of the above markers.

### Detection of different blood indices

The whole blood samples including leucocyte level, hemoglobulin concentration, neutrophil level, lymphocyte level, monocyte level, platelet level were analyzed by BC-6800 fully automatic blood analyzer (Mindray). The serum samples including albumin and CRP were analyzed by Cobas 8000 c702 fully automatic biochemical analyzer.

As for analysis method, laser flow cell technology and fluorescence staining detection technology were performed in the analysis of leucocyte level, neutrophil level, lymphocyte level, monocyte level. Platelet level was analyzed by Sheath DC detection method. And we used bromcresol green method at albumin level while CRP was analyzed by latex agglutination test.

### Statistical analyses

Categorical data are presented as the numbers (percentage); continuous data are presented as the mean (± standard deviation) if normally distributed or as the median (interquartile range) if not normally distributed. Comparisons between two groups were performed using Mann-Whitney U or chi-square tests. The Kaplan-Meier method was used to generate the OS curve, and the survival differences were compared with the log-rank test. A multivariate analysis was performed with the Cox proportional hazards model, and prognostic variables were introduced in the model when the univariate analysis revealed a significance level of *P* <  0.05. The X-tile program (Version 3.1.2, Yale University) was used to calculate the optimal cutoff points for the change rate (α) of each marker. Statistical analyses were performed using SPSS for Windows, version 25.0. All values with P <  0.05 were statistically significant.

## Results

### Inflammatory and nutritional marker changes during NACT

**Fig. 1:**
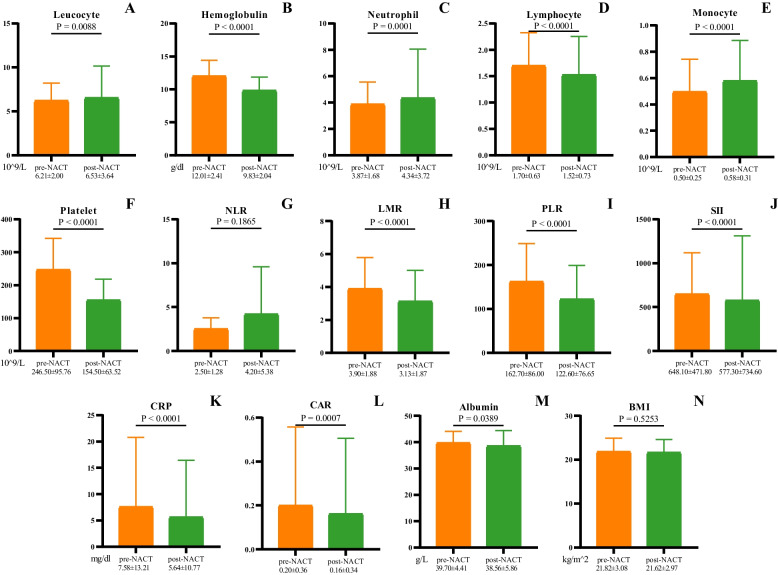
Most numerical data on the inflammatory and nutritional markers changed during NACT except (**G**) neutrophil-to-lymphocyte ratio (NLR) and (**N**) body mass index (BMI). Markers including (**B**) hemoglobulin concentration, (**D**) lymphocyte level, (**F**) platelet level, (**H**) lymphocyte-to-monocyte ratio (LMR), (**I**) platelet-to-lymphocyte ratio (PLR), (**J**) systemic immune-inflammation index (SII), (**K**) C-reactive protein (CRP) level and (**L**) C-reactive protein-to-albumin ratio (CAR), (**M**) serum albumin decreased while (**A**) leucocytes level, (**C**) neutrophils level, and (**E**) monocytes level increased during NACT (*P* < 0.05). Values are presented as mean ± standard deviation

Figure 1 indicates the changes in inflammatory and nutritional markers before and after NACT. No variation was shown only in the NLR levels and BMI in the course of NACT. Most of the inflammatory markers decreased, including lymphocytes, platelets, LMR, PLR, SII, CRP, and CAR, while leucocytes, neutrophils, and monocytes increased after NACT. Regarding the nutritional markers, the hemoglobulin and albumin levels were slightly decreased during NACT.

### The relationship of prognosis and hematologic indexes and BMI after grouping by lab Normal standard in pre-NACT and post-NACT

As the hematologic markers were divided into 2 groups (normal/abnormal), as shown in Fig. S1, 326 patients with normal BMI before NACT had a favorable OS than the other 111 patients (*P* = 0.0106), and 305 patients with normal lymphocyte level after NACT also had a better OS than the other 132 patients (*P* <  0.001).

**Fig. 2:**
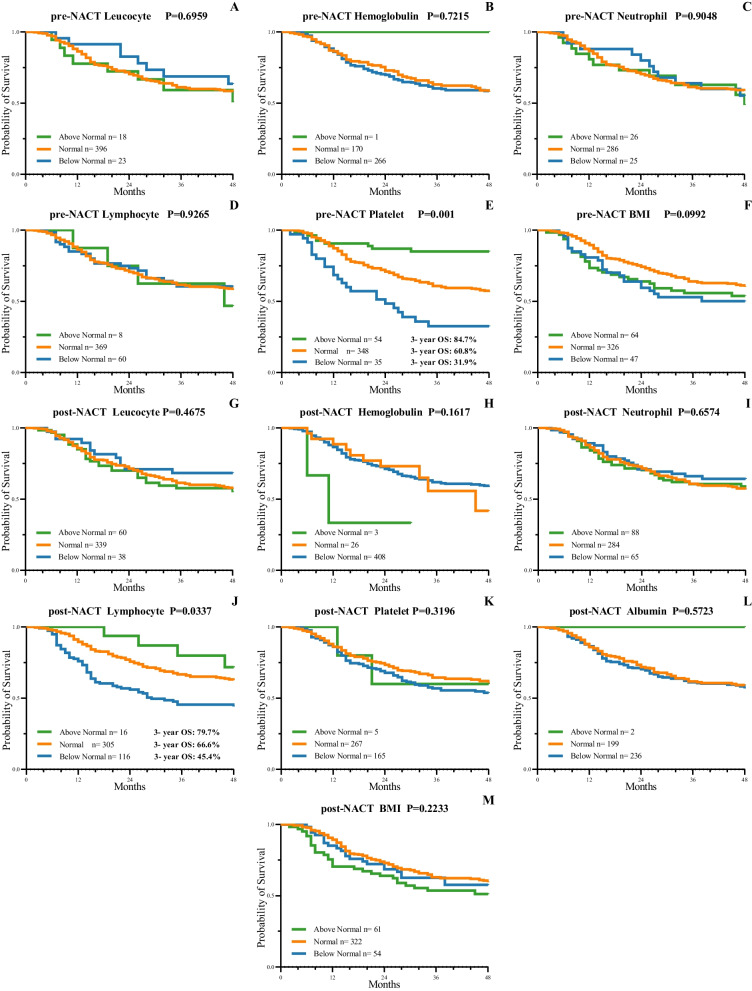
Kaplan–Meier survival curves according to hematologic indexes associated with overall survival (OS) when divided into 3 groups (below/normal/above). OS outcomes according to indexes including leucocyte level, hemoglobulin concentration, neutrophil level, lymphocyte level, platelet level, serum albumin and body mass index (BMI) in pre-NACT (pre-neoadjuvant chemotherapy) (**A-F**) and post-NACT (post-neoadjuvant chemotherapy) (**G-M**). A higher pre-NACT platelet (**E**) and a higher post-NACT lymphocyte (**J**) were both associated with a preferable 3-year OS (*P* < 0.05)

As the hematologic markers were divided into 3 groups (below/normal/above), as shown in Fig. 2, 54 patients whose platelet level before NACT were higher than normal had the best prognosis, and 348 patients whose platelet level were normal had a better OS than the patients whose platelet level were below normal (*P* <  0.001). Moreover, 16 patients whose lymphocyte level after NACT were higher than normal had the best prognosis, and 305 patients whose lymphocyte level were normal had a better OS than the patients whose lymphocyte level were below normal (*P* <  0.001).

With regard to the 3-year survival rate, the patients with normal pre-NACT BMI had a 3-year OS of 63.9%, the patients with abnormal BMI had a 3-year OS of 54.7%, the patients with normal post-NACT lymphocyte level had a 3-year OS of 66.4%, and the patients with abnormal post-NACT lymphocyte level had a 3-year OS of 48.6%.

Moreover, the patients whose pre-NACT platelet level were above normal had the best prognosis, with a 3-year OS of 84.7%, and compared to the patients whose platelet level were below normal, with a 3-year OS of 31.9%, the patients whose platelet level stayed normal had a better prognosis, with a 3-year OS of 60.8%. For post-NACT lymphocyte level, the patients whose lymphocyte level were above normal had the best prognosis, with a 3-year OS of 79.7%, and compared to the patients whose platelet level were below normal, with a 3-year OS of 45.4%, the patients whose platelet level stayed normal had a better prognosis, with a 3-year OS of 66.6%.

### The connection between prognosis and the changes in hematologic indexes and BMI during NACT

**Fig. 3:**
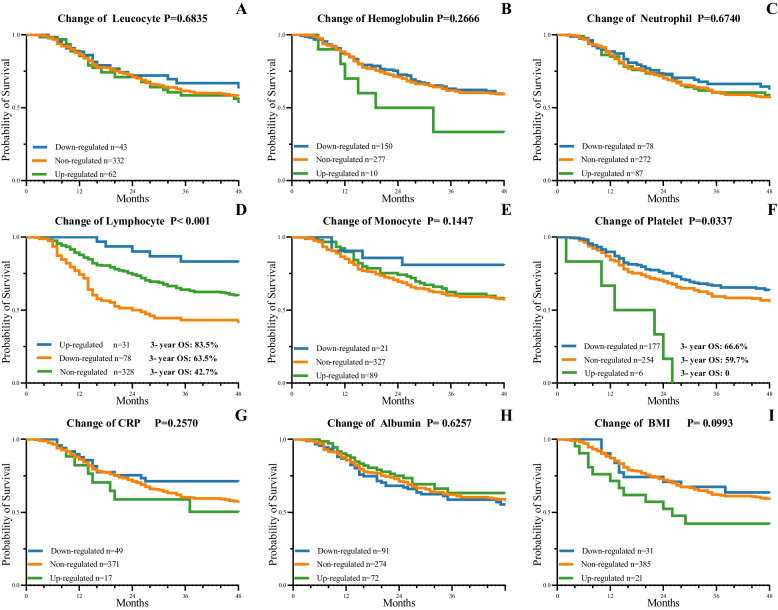
Kaplan–Meier survival curves of the change in hematologic indexes (**A-I**) including leucocyte level, hemoglobulin concentration, neutrophil level, lymphocyte level, monocyte level, platelet level, C-reactive protein (CRP) level, albumin level, body mass index (BMI) during NACT (neoadjuvant chemotherapy). The up-regulated lymphocyte and down-regulated platelet showed positive association with the 3-year overall survival rate (*P* < 0.05)

As Fig. 3 shows, the change in the hematologic indexes, including hemoglobulin, platelet and lymphocyte level and BMI during NACT also had a significant correlation with OS. The patients with up-regulated hemoglobulin or up-regulated BMI both have an adverse prognosis compared with the other patients.

In addition, those whose platelet level decreased during NACT had a better OS than those whose platelet level increased or remained unchanged. All of the patients with higher platelet level after NACT died within 3 years. Conversely, the patients with higher lymphocyte level after NACT had a preferable prognosis than the patients with invariant or lower lymphocyte level.

### The relationship of prognosis and systemic inflammatory and nutritional markers’ change rate after grouping by X-tile

**Fig. 4:**
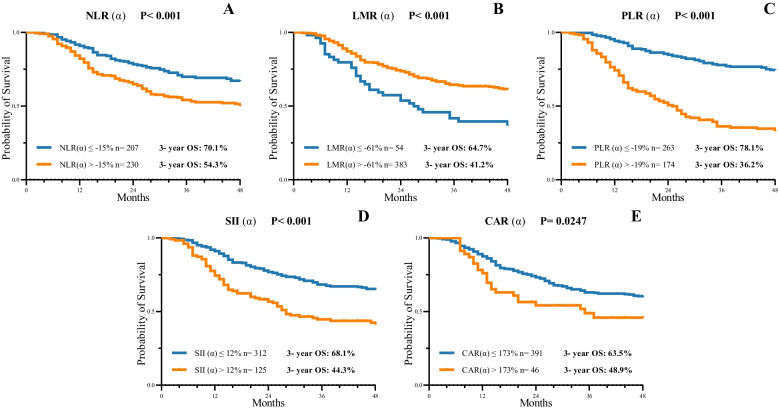
Kaplan–Meier survival curves of the changes in systemic inflammatory and nutritional markers (**A-E**) including neutrophil-to-lymphocyte ratio (NLR), lymphocyte-to-monocyte ratio (LMR), platelet-to-lymphocyte ratio (PLR), systemic immune-inflammation index (SII), C-reactive protein-to-albumin ratio (CAR), which were selected through X-tile. NLR (α) ≤ − 15%, LMR (α) > − 61%, PLR (α) ≤ − 19%, SII (α) ≤ 12%, CAR (α) ≤ 173% were all associated with a preferable 3-year OS (*P* < 0.05)

The change rates of NLR, LMR, PLR, SII, and CAR were associated with OS after grouping by cutoff values derived from X-tile (*P* <  0.05). Figure 4 shows that the optimal cutoff points for NLR, LMR, PLR, SII, and CAR change rate (α) were − 15%, − 61%, − 19%, 12%, and 173%, respectively, according to the X-tile plots. The patients with PLR (α) > − 19% had a favorable OS compared to the patients whose descent rate was more than 19%. Moreover, such differences were also observed with changes in the NLR (*P* <  0.001), LMR (*P* < 0.001), SII (*P* < 0.001) and CAR (*P* = 0.0247).

### Several factors were associated with prognosis in gastric cancer patients

As indicated in Table 1, the univariate analysis showed that differentiation, TRG, pre-NACT BMI, pre-NACT platelet level, post-NACT lymphocyte level, change in lymphocyte level, change in platelet level, LMR change rate (α), PLR change rate (α), SII change rate (α), and CAR change rate (α) were closely related to OS (*P* < 0.05).

**Table 1:** Univariate and multivariate analyses of OS in advanced gastric cancer patients

Variable	Univariate			Multivariate		
	HR	95% CI	***P***	HR	95% CI	***P***
**Sex**						
Male	Ref.					
Female	1.258	0.910–1.739	0.165			
**Age**						
≤ 60	Ref.					
> 60	1.102	0.818–1.484	0.523			
**Tumor location**						
Upper	Ref.					
Middle	1.106	0.721–1.697	0.645			
Lower	1.134	0.947–1.357	0.170			
**NACT Cycle**						
< 3	Ref.					
≥ 3	1.061	0.928–1.211	0.388			
**Surgery**						
Open	Ref.					
Laparoscope	0.923	0.534–1.594	0.773			
**Differentiation**						
Poor	Ref.			Ref.		
Well- moderate	0.386	0.223–0.666	**0.001**	0.452	0.259–0.788	**0.005**
**Operation mode**						
Proximate GR	Ref.					
Distal GR	1.468	0.204–10.559	0.703			
Total GR	1.212	0.453–3.246	0.702			
**TRG**						
Tumor residue	Ref.			Ref.		
Tumor regression	0.603	0.400–0.909	**0.016**	0.629	0.413–0.958	**0.031**
**pre- NACT BMI**						
Normal	Ref.			Ref.		
Unnormal	1.412	1.021–1.952	**0.037**	1.304	0.931–1.827	0.122
**pre- NACT PLT**						**0.011**
Normal	Ref.			Ref.		
Below normal	2.097	1.350–3.258	**0.001**	1.283	0.736–2.236	0.379
Above normal	0.558	0.391–0.797	**0.001**	0.337	0.161–0.706	**0.004**
**post- NACT Lym**						0.555
Normal	Ref.			Ref.		
Below normal	1.894	1.389–2.585	**< 0.001**	1.718	0.553–5.340	0.350
Above normal	0.611	0.474–1.287	0.333	0.866	0.513–1.464	0.592
**LMR**						
α > −61%	Ref.			Ref.		
α ≤ −61%	2.006	1.373–2.930	**< 0.001**	1.279	0.823–1.986	0.273
**PLR**						
α ≤ −19%	Ref.			Ref.		
α > −19%	3.914	2.872–5.334	**< 0.001**	3.193	2.194–4.649	**< 0.001**
**SII**						
α > 12%	Ref.			Ref		
α ≤ 12%	2.154	1.593–2.913	**< 0.001**	1.016	0.704–1.468	0.931
**CAR**						
α > 173%	Ref.			Ref.		
α ≤ 173%	1.625	1.056–2.500	**0.027**	1.375	0.877–2.154	0.165
**Change of Lym**						0.099
Up-regulated	Ref.			Ref.		
Non-regulated	2.775	1.135–6.783	**0.025**	2.550	0.918–7.086	0.073
Down-regulated	5.293	2.100–13.342	**< 0.001**	3.576	1.114–11.478	**0.032**
**Change of PLT**						0.156
Down-regulated	Ref.			Ref.		
Non-regulated	0.774	0.567–1.058	0.109	1.364	0.938–1.983	0.104
Up-regulated	4.384	1.917–10.028	**< 0.001**	1.710	0.658–4.446	0.271

*NACT* neoadjuvant chemotherapy, *GR* gastrectomy, *TRG* tumor regression grade, *BMI* body mass index, *PLT* platelet, *Lym* lymphocyte, *LMR* lymphocyte-to-monocyte ratio, *PLR* platelet-to-lymphocyte ratio, *SII* systemic immune-inflammation index, *CAR* C-reactive protein-to-albumin ratio

The multivariate analysis with the Cox proportional hazards model indicated that differentiation (*P* = 0.005), TRG (*P* = 0.031), and PLR change rate (α) (*P* < 0.001) were independent prognostic indicators. The patients whose PLR (α) was more than − 19% had a 3.193-fold (95% CI: 2.194–4.649) higher risk of death (*P* < 0.001) than the others.

## Discussion

In view of the critical situation regarding GC in China, exploring more markers to predict prognosis accurately is particularly important. In addition, real-time monitoring of some indicators that can predict the prognosis of GC patients could provide a basis for the dynamic adjustment of treatment regimens.

Our study evaluated the predictive value of the changes in inflammatory and nutritional markers in the perioperative period (the variation between the preoperative and pre-NACT levels) on long-term outcomes in patients with advanced GC. First, we confirmed that most of the inflammatory and nutritional markers changed significantly (*P* < 0.05) after several cycles of NACT, except NLR and BMI. Furthermore, after dividing the patients into several groups according to the standard range of peripheral blood tests from the database, we found that pre-NACT BMI, pre-NACT platelet level and post-NACT lymphocyte level were associated with prognosis by the Kaplan–Meier method. With regard to the changes in the hematologic indexes, down-regulated lymphocyte level and up-regulated platelet level led to a poor prognosis. For systemic inflammatory markers, we divided the patients into two groups according to the critical value of some possibly significant markers’ rate of change (α) exported by X- tile separately. We found that LMR (α < − 61%), PLR (α ≤ − 19%), SII (α ≤ 12%) and CAR (α > 173%) had a positive correlation with good prognosis. Whereas in the multivariate analysis, only the platelet level before NACT, and the systemic inflammatory marker PLR were critical factors associated with the OS of GC patients. Obviously, the changes in the platelet and lymphocyte level subsequently led to the tendencies of PLR, which combines platelet and lymphocyte level. In addition, the patients with poor tumor differentiation experienced adverse OS. Interestingly, TRG was intimately related to OS, and the patients with a better pathological response to NACT had a better prognosis.

When microscopic examination was applied to tumors, a great number of studies concentrated on the microenvironment of tumors and immunity and identified several novel and meaningful concepts, such as the “tumor microenvironment” or “immune microenvironment” [15]. Although immunity and inflammation constitute the basic characteristics of the tumor microenvironment, their exact relationship remains vague [16]. As we currently know, acute inflammation eliminates cancer cells by inspiring an antitumor immune response, while chronic inflammation is relevant to immunodepression, thus offering a microenvironment for tumor development [17]. Along with the fact that inflammation is strongly associated with all stages of development and that malignant progression of cancer has been verified in a vast majority of cancers, studies focusing on the effects of inflammatory markers on tumors have continued to emerge [18]. Numerous studies have shown that several inflammatory markers are related to the prognosis of gastrointestinal cancer, especially CRP, LMR PLR and NLR [19–23]. For advanced gastric cancer patients who have undergone preoperative neoadjuvant chemotherapy and radical resection, an associated study of Peking University Cancer Hospital reported that elevated NLR before NACT, preoperative anemia and larger change value of LMR implied an adverse prognosis [24]. Our study also supports the idea that there existed a relationship between changes in the NLR and LMR during NACT and survival outcomes [19]. However, little variation was shown in NLR levels in the course of NACT. Regarding nutritional markers in a recent study, many composite biomarkers that contain albumin, such as CAR (C-reactive protein/albumin), AFR (albumin/fibrinogen), and AGR (albumin/globulin), were reported to be correlated with the OS of gastric patients [23, 25]. In the present study, we only analyzed CAR(α) and presented its significant relationship with prognosis, while we could not demonstrate any correlation with albumin. Regarding PLR in a recent study, many studies have reported that it is correlated with the OS of cancer patients, such as hepatocellular carcinoma, ovarian carcinoma, and advanced colorectal cancer [26, 27]. Furthermore, research from King Hussein Cancer Center (Amman, Jordan) reported that high PLR is associated with distant metastases on presentation in gastric cancer [28]. Even more interesting, Kagoshima University Hospital (Kagoshima, Japan) combined the NLR and PLR to draw a grading rule and eventually thought the NLR-PLR score was an independent prognostic factor for the prediction of OS [29]. Taking comprehensive aclevel of the above factors, we considered PLR, which combines the platelet and lymphocyte level, as a representative and meaningful inflammatory and nutritional marker. Many studies similarly recommended predictive models containing inflammatory, nutritional and tumor markers in GC patients. The research of Nanjing First Hospital supposed that the predictive model combining NLR and CA199 markers could accurately predict the personalized survival of surgical GC patients [23]. West China Hospital combined with Hai Kou Hospital claimed that body mass index-lymphocyte-carbohydrate antigen 19–9 (BLC) was an independent prognostic predictor for surgical GC patients [21].

Since the present research verified that surgery and systemic chemotherapy were reliable treatments for advanced gastric cancer patients, perioperative chemotherapy was extremely meaningful to both patients and surgeons [30]. To decide the number of cycles of perioperative chemotherapy and choose an appropriate operational opportunity, clinicians desperately need a credible reference. Our study found several inflammatory and nutritional markers that made it possible to construct a predictive model and to facilitate decision making.

Our study also had some limitations. First, we only used a retrospective design at a single institution and analyzed a small number of patients. Second, we unsured PSM (propensity score matching) between groups to reduce confounding bias. Third, PLR as a systemic inflammatory marker has not been validated internally or externally. Finally, we hope to build a more accurate predictive model by combining more factors, such as tumor markers and postoperative pathology.

In conclusion, we systematically analyzed each of the inflammatory and nutritional marker and changes in these markers during NACT in 437 advanced GC patients. We found that NACT could significantly change several inflammatory and nutritional markers in the perioperative period except for NLR and BMI. Pathological features, including differentiation and TRG, were associated with prognosis. Regarding inflammatory and nutritional markers, the platelet level before NACT and the lymphocyte changes during NACT were correlated with long-term survival outcomes among patients with advanced gastric cancer. The systemic inflammatory marker PLR may be a reliable marker for predicting prognosis.

## Supplementary Information

**Additional file 1: Supplementary Table 1.** Clinical–pathological data of included patients. **Supplementary Fig. 1.** Kaplan–Meier survival curves according to hematologic indexes associated with overall survival (OS) outcomes when divided into 2 groups (normal/abnormal). OS outcomes according to indexes including leucocyte level, hemoglobulin concentration, neutrophil level, lymphocyte level, monocyte level, platelet level, C-reactive protein (CRP) level, serum albumin, and body mass index (BMI) in pre-NACT (pre-neoadjuvant chemotherapy) (A-I) and post-NACT (post-neoadjuvant chemotherapy) (J-R). Normal pre-NACT BMI (I) and normal pre-NACT lymphocyte (M) showed positive association with the 3-year overall survival rate (*P* < 0.05).

## Data Availability

Due to the privacy of patients, the data related to patients cannot be available for public access but can be obtained from the corresponding author on reasonable request approved by the institutional review board of all enrolled centers.

## Abbreviations

NACT: Neoadjuvant Chemotherapy
R0: Radical Resection
TNM: Tumor Node Metastasis
OS: Overall Survival
GC: Gastric Cancer
TIME: Tumor Immune Microenvironment
TRG: Tumor Regression Grade
CRP: C-reactive Protein Level
NLR: Neutrophil-to-lymphocyte Ratio
LMR: Lymphocyte-to-monocyte Ratio
PLR: Platelet-to-lymphocyte Ratio
SII: Systemic Immune-inflammation Index
CAR: Protein-to-albumin Ratio
BMI: Body Mass Index
GR: Gastrectomy
