# Supplementary material for: Prognostic significance of inflammatory and nutritional markers in perioperative period for patients with advanced gastric cancer
Source: BMC Cancer. 2023 Jan 3;23:5. doi: 10.1186/s12885-022-10479-6 (PMC9808945; doi:10.1186/s12885-022-10479-6)
Supplement: Supplementary file 1 — Additional file 1: Supplementary Table 1. Clinical–pathological data of included patients. Supplementary Fig. 1. Kaplan–Meier survival curves according to hematologic indexes associated with overall survival (OS) outcomes when divided into 2 groups (normal/abnormal). OS outcomes according to indexes including leucocyte level, hemoglobulin concentration, neutrophil level, lymphocyte level, monocyte level, platelet level, C-reactive protein (CRP) level, serum albumin, and body mass index (BMI) in pre-NACT (pre-neoadjuvant chemotherapy) (A-I) and post-NACT (post-neoadjuvant chemotherapy) (J-R). Normal pre-NACT BMI (I) and normal pre-NACT lymphocyte (M) showed positive association with the 3-year overall survival rate (P < 0.05). [file 12885_2022_10479_MOESM1_ESM.docx]

**Supplementary Table 1.** Clinical–pathological data of included patients.

| **Variable** | **Cases, n(%)** |
| --- | --- |
| **Sex** |  |
| Male | 325 (74.37) |
| Female | 112 (25.63) |
| **Age** |  |
| >60 | 200 (45.77) |
| ≤60 | 237 (54.23) |
| **NACT Cycle** |  |
| ≥3 | 240 (54.92) |
| <3 | 197 (45.08) |
| **Tumor location** |  |
| Upper | 126 (28.83) |
| Middle | 103 (23.57) |
| Lower | 208 (47.60) |
| **Differentiation** |  |
| Poor | 367 (83.98) |
| Well- moderately | 70 (16.02) |
| **TRG** |  |
| Tumor regression | 91 (20.82) |
| Tumor residue | 346 (79.18) |
| **Surgical method** |  |
| Open | 398 (91.08) |
| Laparoscope | 39 (8.92) |
| **Type of resection** |  |
| Proximate gastrectomy | 4 (0.92) |
| Distal gastrectomy | 180 (41.19) |
| Total gastrectomy | 253 (57.89) |

Values are presented as number (%). NACT, neoadjuvant chemotherapy; TRG, tumor regression grade.

**Clinicopathological Characteristics of the Patients**

As Table 1 shows, there were 325 (74.37%) male patients and 112 (25.63%) female patients. The median age was 61.0 (21.0~80.0) years; 200 (45.77%) patients were aged over 60 years; and 237 (54.23%) patients were under 60 years. A total of 240 (54.92%) patients accepted 3 rounds of NACT or more, while 197 (45.08%) patients accepted fewer than 3 rounds. The number of patients with tumor locations in the upper, middle, and lower regions were 126 (28.83%), 103 (23.57), and 208 (47.60%), respectively. In terms of operation, 398 (91.08%) patients underwent open surgery, and only 39 (8.92%) patients underwent laparoscope surgery. Moreover, proximate gastrectomy (GR) was only performed on 4 (0.92%) patients, while distal GR and total GR were performed on 180 (41.19%) patients and 253 (57.89%), respectively.

Regarding the pathological features, tumor regression (TRG0 and TRG1) was acquired in 91 (20.82%), and tumor residue (TRG2 and TRG3) was acquired in 346 (79.18%). With regard to tumor differentiation, 367 (83.98%) patients had poor differentiation, and 70 (16.02%) patients had well- moderate differentiation.


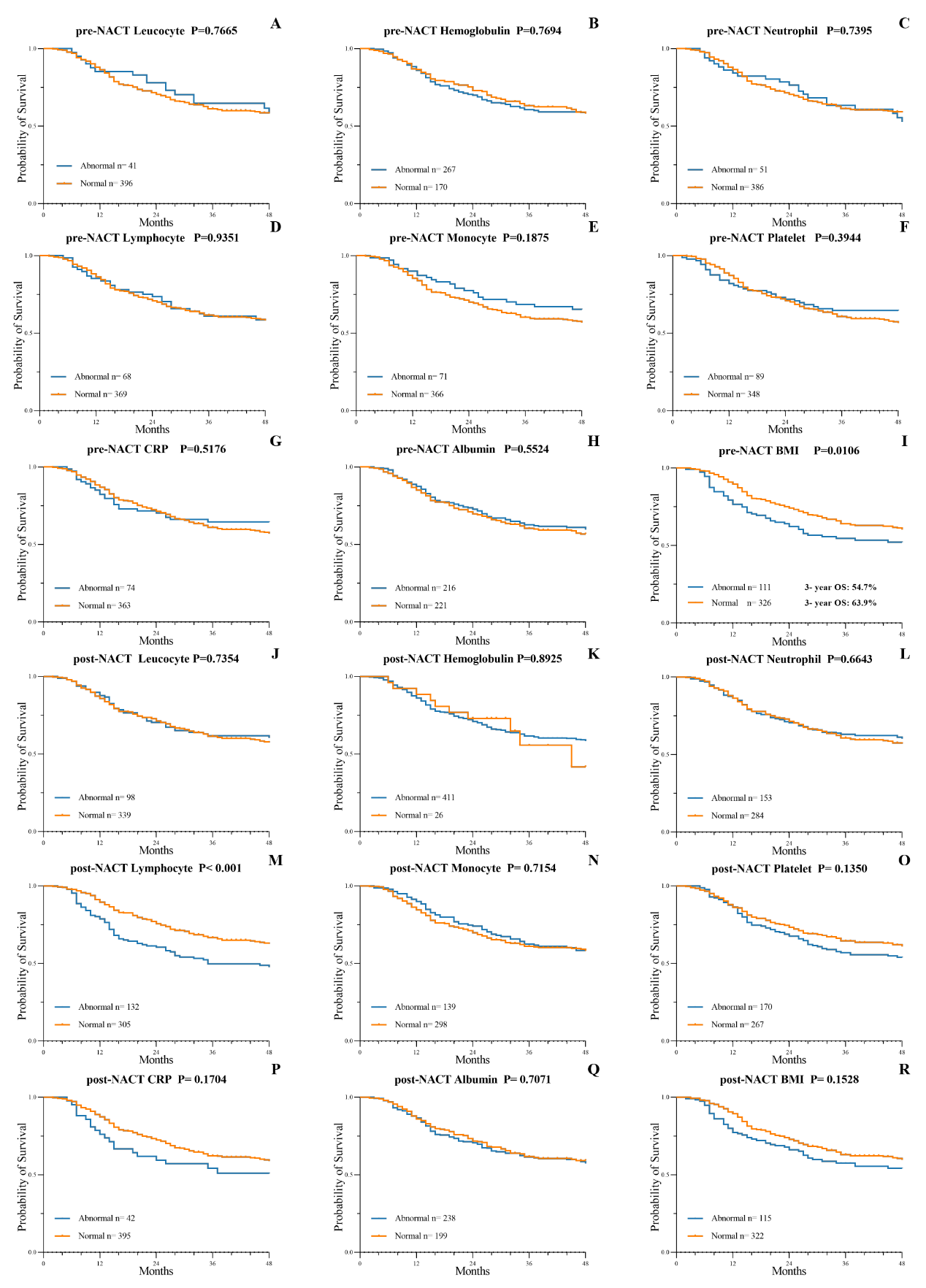


**Supplementary Figure 1**. Kaplan‒Meier survival curves according to hematologic indexes associated with overall survival (OS) outcomes when divided into 2 groups (normal/abnormal). OS outcomes according to indexes including leucocyte level, hemoglobulin concentration, neutrophil level, lymphocyte level, monocyte level, platelet level, C‑reactive protein (CRP) level, serum albumin, and body mass index (BMI) in pre-NACT (pre-neoadjuvant chemotherapy) (A-I) and post-NACT (post-neoadjuvant chemotherapy) (J-R). Normal pre-NACT BMI (I) and normal pre-NACT lymphocyte (M) showed positive association with the 3-year overall survival rate (P<0.05).
